# Integrative Analysis of Proteomics and Transcriptomics of *Longissimus dorsi* with Different Feeding Systems in Yaks

**DOI:** 10.3390/foods12020257

**Published:** 2023-01-06

**Authors:** Xiaoming Ma, Xian Guo, Yongfu La, Xiaoyun Wu, Min Chu, Pengjia Bao, Ping Yan, Chunnian Liang

**Affiliations:** 1 Animal Science Department, Lanzhou Institute of Husbandry and Pharmaceutical Sciences, Chinese Academy of Agricultural Sciences, Lanzhou 730050, China; 2 Key Laboratory of Animal Genetics and Breeding on Tibetan Plateau, Ministry of Agriculture and Rural Affairs, Chinese Academy of Agricultural Sciences, Lanzhou 730050, China; 3 Key Laboratory for Yak Genetics, Breeding, and Reproduction Engineering of Gansu Province, Lanzhou 730050, China

**Keywords:** yak, *Longissimus dorsi* muscle, meat quality, RNA sequencing, TMT proteomics

## Abstract

Yaks (*Bos grunniens*) are a critical livestock breed in the plateau region, and changing the feeding system of yaks can significantly improve their growth performance. The effects of different feeding regimes on the growth performance and meat quality of yaks were comprehensively compared here. The transcriptome and proteome of the *Longissimus dorsi* muscle were determined using RNA-seq and Tandem Mass Tag (TMT) techniques. Indoor feeding significantly improved the growth performance (such as the average daily gain and carcass weight) and meat quality characteristics compared with traditional grazing feeding. In the grazing (Group G) vs. in-house fed group (Group HF) comparison, 40 differentially expressed genes/differentially abundant proteins exhibited the same mRNA and protein expression trends. These genes were associated with collagen binding, the lipoxygenase pathway, and the arachidonic acid metabolic process. Parallel reaction monitoring verified whether the TMT results were reliable. Moreover, some pathways, such as the AMPK signaling pathway, FoxO signaling pathway, PPAR signaling pathway, and fatty acid metabolism, were significantly enriched. These results expand our knowledge about meat quality in yaks and provide practical information and more evidence for further insight into the biological mechanisms underlying meat quality traits.

## 1. Introduction

In the Qinghai–Tibet Plateau and its adjacent pastoral areas, yak (*Bos grunniens*), known as multi-functional livestock, is an indispensable animal breed because it is the primary source of meat, milk, and fur and an essential means of production and living for local herders. Currently, yaks are mainly raised through grazing-based systems. Because of the unique climatic characteristics of its habitat, the cold season is incredibly long (from the beginning of October to the end of May). In the cold season, herbage yield and nutritional quality are significantly reduced, and therefore, yaks exhibit severe nutritional deficiency. To cope with the situation, they only consume the fat accumulated in the warm season (from early June to late September) and maintain their primary metabolism. Consequently, the weight of yaks decreases by 25% after each cold season, and the feeding cycle becomes more extended, thereby significantly decreasing the benefits of breeding. The seasonal fattening technique of yak in semi-farming and semi-grazing areas has recently been considered among the crucial ways of shortening the feeding cycle, improving the production performance, and enhancing the meat quality of yaks. The popularization and application of this technology can not only shorten the time for a yak to pasture and increase herders’ income but also improve grazing. This would reduce the decline of pastures [1,2].

Skeletal muscle provides security for mammals’ movement, and the surrounding tissue exhibits glucose and fatty acid (FA) oxidation, providing energy to important tissues [3]. Muscles are the primary sites where chemical energy is converted into mechanical energy, which is required for muscle contraction and maintenance of muscle functional integrity. In the muscle, the complete oxidation of carbohydrates to CO_2_ and H_2_O requires oxygen and produces energy. Most energy is obtained through the addition of a phosphate group to another molecule with two phosphate groups [4]. Glycogen, the main stored carbohydrate in muscle fibers, is present as a single particle or lump in the sarcoplasm between the myofibrils and cell membrane. It accounts for approximately 0.5%–1.3% of muscle weight. Phosphorylase and other enzymes (pyruvate kinase and phosphofructokinase-1), in combination, lead to glycogen breakdown in muscle cells [5]. Studies have shown that muscle energy is directly related to meat quality and feeding regimes [6,7]. Nutritional (protein) intake is among the essential factors determining animal meat quality. Muscle nutrients correlate with meat quality [8]. However, knowledge about muscle development and changes in feeding systems in yaks is limited. A comprehensive multi-omics analysis offers necessary insights into muscle development while facilitating increased productivity of yaks.

Studies have shown that fattening can significantly improve slaughter performance and the meat quality of livestock [9,10,11]. However, reports on improving meat quality through yak fattening are few. We here analyzed the growth performance and meat and carcass traits of yaks under two feeding modes by using TMT proteome and RNA-seq technology. The research results provide a scientific basis for large-scale fattening and integrated demonstration of high-quality production technology for yak meat.

## 2. Materials and Methods

All yaks were strictly handled according to the good animal practices that complied with the Animal Ethics Procedures and Guidelines of the People’s Republic of China. The study approval was from the Animal Administration and Ethics Committee of Lanzhou Institute of Husbandry and Pharmaceutical Sciences of the Chinese Academy of Agricultural Sciences (Permit No. 2019-002).

### 2.1. Animals, Feeding Regimes, and Weight Determination

Twelve 4-year-old male Ashdan yaks were selected from the Datong Cattle farm in Qinghai Province and randomly divided (6 in each group) into grazing (Group G) and in-house fed groups (Group HF). The experiment was conducted from 5 April to 2 October 2019 on the same farm. Group G was allowed to graze freely within the pasture located on Datong yak farm, and Group HF was fed in-house. The pre-trial period lasted 10 days, and the trial period for 170 days. The diet consisted of concentrate, which comprised commonly used feed materials, and crude feed (contained oat hay and alfalfa hay). Table 1 presents the composition and nutrient levels of the experimental diet. All yaks were treated with deinsectization during pre-feeding. Yaks in the HF group were fed once in the morning and once in the evening and allowed free access to drinking water. The concentrate feeding status was observed and recorded.

### 2.2. Tissue Collection and Measurements

At the end of the feeding experiment, all yaks were water-deprived between 08:30 a.m. and 10:30 a.m. The yaks were slaughtered 24 h after fasting. The carcass and live weights before slaughter were recorded, and the eye muscle area and slaughter rate were measured [12]. The pH value, meat color, shear force, cooking loss, and drop loss rate of the 12th to 13th intercostal *Longissimus dorsi* (LD) muscle were measured at 45 min and 24 h [13]. Meanwhile, 6 yak LD muscle samples were randomly selected from each experimental group. The samples were cut into small pieces (diameter of 1–3 cm^3)^, placed in cryo-storage tubes, transported to the laboratory using dry ice, and stored in a refrigerator at −80 °C for long-term storage.

### 2.3. RNA-Seq Analysis

Total RNA was extracted from yak LD using the TRIzol reagent (Invitrogen, Carlsbad, CA, USA). RNA quality was detected using the Agilent 2100 bioanalyzer (Agilent Technologies, Santa Clara, CA, USA), and the RNA was subjected to 1% agarose gel electrophoresis to assess RNA integrity. Subsequent analyses were performed on samples with an RNA Integrity Index (RIN) of ≥7. Libraries were constructed using TruSeq Stranded Total RNA with Ribo-Zero Gold and sequenced on an Illumina sequencing platform (HiSeqTM 2500), and 150-bp/125-bp paired-end reads were generated.

The raw reads generated during high-throughput sequencing were fastq-format sequences. To obtain high-quality reads that can be used for subsequent analysis, raw reads need to be further quality-filtered. First, Trimmomatic software [14] was used to remove adapters and low-quality bases, and N-bases or low-quality reads (LEADING: 3 TRAILING: 3ILLUMINACLIP: TruSeq3-PE-2.fa: 2: 30: 10: 8: true Sliding window: 4:15 MINLEN:50) were filtered out. Finally, high-quality clean reads were obtained. These clean reads were aligned to the reference genome (GCF_000298355.1_BosGru_v2.0) of BosGru_v2.0 by using hisat2 [15]. The samples should be evaluated by genome and gene alignment. Cufflinks software [16] was used to calculate FPKM values for each protein-coding gene. The counts were normalized using the estimateSizeFactors function of the DESeq R package [17], and the *p*-value and fold change values were calculated using the nbinomTest function for differential comparison. Differential transcripts with *p* ≤ 0.05 and |log2FC| > 1 were selected as DEGs.

### 2.4. Protein Digestion and TMT Labeling

The lysate was added to each LD sample and centrifuged at 2000× *g* at 4 °C for 10 min. Trichloroacetic acid was added to the supernatant. The mixture was allowed to stand at 4 °C, centrifuged at 12,000× *g* for 3 min, washed with acetone, precipitated, redissolved with 8 M urea, and quantified with the BCA protein kit. Trypsin was added and digested overnight at 37 °C. The trypsin-hydrolyzed peptide was desalted with Strata XC18 and freeze-dried in a vacuum. Peptides were dissolved with 0.5 M TEAB and labeled according to the instructions of the TMT kit.

The peptides were fractionated using high-pH reverse-phase high-performance liquid chromatography (HPLC). Subsequently, the peptides were combined into 18 components, and the combined components were freeze-dried in a vacuum. The peptides were dissolved using liquid chromatography mobile phase A and separated using the EASY nLC1000 ULTRA HPLC system. The peptides were separated using an ultra-performance liquid phase system, injected into an NSI ion source for ionization, and then analyzed through mass spectrometry. Secondary mass spectrometry data were retrieved using Maxquant (VL.5.2.8). The identified DAPs were calculated using the fold change (FC) and *p*-value based on the LFQ value and screened according to the protein quantification method. A global false discovery rate (FDR) of <1% was used, and a minimum of 2 peptides was required for the peptide set considered for quantification. The *p*-values for trusted proteins calculated using the t-test showed significant differences between groups (fold change ≥ 1.2 as upregulate, fold change < 1/1.2 as downregulate, and *p*-value < 0.05). Proteome Discover 2.4 (Thermo Fisher Scientific, Waltham, MA, USA) and the Uniprot protein database (www.Uniprot.com, accessed on 11 October 2022.) were used for analysis 

The DAPs/DEGs were screened for Gene Ontology (GO) functional annotation and Kyoto Encyclopedia of Genes and Genomes (KEGG) signal pathway analysis. The functions and KEGG signal pathway were enriched with differential proteins screened.

### 2.5. Multi-Omics Joint-Level Analysis (Transcriptome and Proteome)

Understanding the correlation between yak muscle transcriptomics and proteomics data can further help comprehend the post-transcriptional and post-translational regulatory mechanisms of genes. The association analysis included the determination of the association between gene and protein expression. Using the transcriptome and proteome data and sharing DEGs/DAPs, we constructed the protein-protein interaction (PPI) network and obtained its virtual node. For the citation analysis, *p* < 0.05 was specified as the screening condition for a significant difference.

### 2.6. Target Analysis Using Parallel Reaction Monitoring

To verify the reliability of proteomics data, parallel reaction monitoring (PRM) was used to verify the DAPs. PRM is a high-resolution, high-precision ion monitoring technology based on MS analysis and can selectively and quantitatively detect target proteins and peptides. According to preliminary proteomics results, peptide information suitable for PRM analysis was imported into XcaliburTM (Thermo Fisher Scientific, Waltham, MA, USA) software for analysis. For chromatographic separation using an HPLC system, 200 fmol of the standard peptide (PRTC: GILFVGSGVSGGEEGAR) was added to each sample. PRM mass spectrometry was performed using q-executive HF MS (Thermo Fisher Scientific, Waltham, MA, USA), and the results were analyzed using Skyline 3.5.0 [18].

### 2.7. Statistical Analyses

The measured growth performance and meat and carcass data were analyzed using the *t*-test in GraphPad Prism 8, and the results are expressed as mean ± standard deviation. *p* < 0.05 was used as the criterion for a significant difference.

## 3. Results

### 3.1. Characterization of Growth Performance and Meat and Carcass Traits

As shown in Table 2, compared with the grazing group, the final body weight, average daily gain, carcass weight, slaughter rate, net meat weight, bone weight, meat–bone ratio, and eye muscle area were significantly increased in the HF group (*p* < 0.01). After 170 days of fattening, the average body weight of male yaks in the fattening group was 377.17 kg; they were 70.34 kg heavier than yaks in the grazing group. The average carcass weight of the fattening group was 83.76 kg higher than that of the grazing group, which indicated that fattening could significantly improve the growth performance traits of Ashdan yak. Table 3 presents the meat quality of yak LD in two feeding modes. The pH at 45 min and 24 h, cooking loss, and shear force of LD muscles differed significantly (*p* < 0.01) between the two groups, whereas no difference in the muscle color index was observed between the two groups.

### 3.2. Overall Statistics for Transcriptomic Analysis

**Figure 1:**
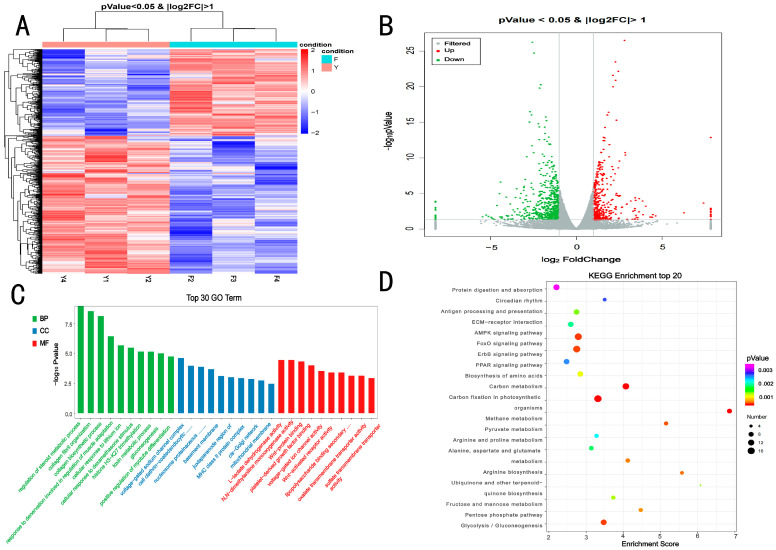
The number of differentially expressed mRNAs in (control/experiment) Group G vs. Group HF comparisons. (**A**) Hierarchical clustering of DEGs, (**B**) Volcano plot of DEGs, (**C**) GO term enrichment for DEGs in the Group G vs. Group HF comparisons, (**D**) KEGG term enrichment for DEGs in the Group G vs. Group HF comparisons.

F2, F3, and F4 served as replicate libraries for Group G, and Y1, Y2, and Y4 served as replicate libraries for Group HF. In the transcriptomic analysis, 104,747,005 and 112,584,959 high-quality clean reads were obtained from Group G and Group HF, respectively. A total of 99,091,520 (Group G) and 108,294,774 (Group HF) clean reads were mapped to the BosGRU_v2.0 genome with mapping ratios of 94.59% and 95.25%, respectively. Among the total mapped reads, 90.69% (Group G) and 90.62% (Group HF) were unique matches to the BosGRU_v2.0 genome, and Q30% values in all libraries exceeded 93.85%, indicating that the clean reads were of high quality (Table S1). The sequencing quality was sufficiently high for the subsequent quantitative analysis. Through transcriptome analysis, 15,242 genes were identified in the two groups. Overall, 1003 DEGs (387 upregulated and 616 downregulated genes) were obtained from the two groups (Group G vs. Group HF; Figure 1A). Moreover, hierarchical clustering results revealed that the expression profiles of genes from the two groups were significantly different (Figure 1A,B).

GO enrichment analysis was performed to identify significantly enriched GO terms among the DEGs. At the transcriptomic level, DEGs detected in the Group G vs. Group HF comparison were significantly (*p* < 0.05) enriched for 650 GO terms (Table S2) and were mainly distributed in the terms regulation of the steroid metabolic process, cellular response to the dexamethasone stimulus, response to denervation involved in the regulation of muscle adaptation, MHC class II protein complex, Z disc, voltage-gated sodium channel complex, NADP binding and Wnt-protein binding (Figure 1C). The KEGG enrichment analysis was conducted to identify significantly enriched KEGG terms among the DEGs. At the transcriptomic level, DEGs detected in the Group G vs. Group HF comparison were significantly (*p* < 0.05) enriched for 63 KEGG terms (Table S2) and were mainly distributed in the terms AMPK signaling pathway, glycolysis/gluconeogenesis, FoxO signaling pathway, PPAR signaling pathway, and ECM–receptor interaction (Figure 1D).

### 3.3. Overall Statistics for Proteomics Analysis

**Figure 2:**
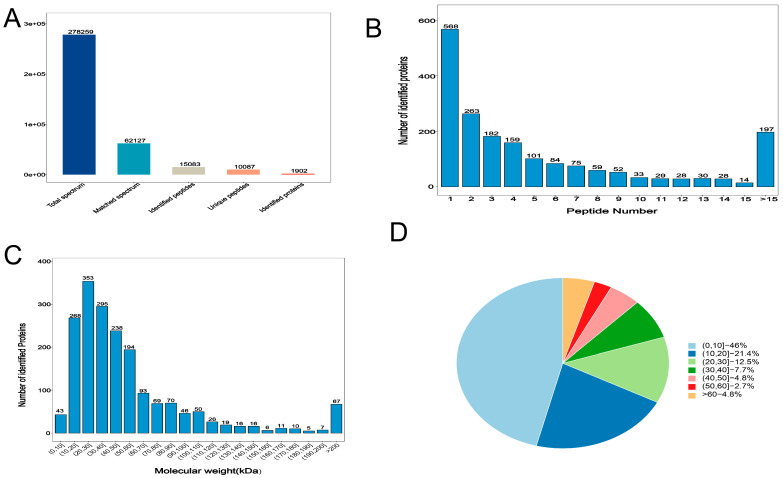
Identification and quantitative evaluation of proteins. (**A**) Identified spectra, peptides, and proteins. (**B**) Peptide quantity distribution (**C**) Protein molecular weight distribution (**D**) Distribution of protein sequence coverages.

**Figure 3:**
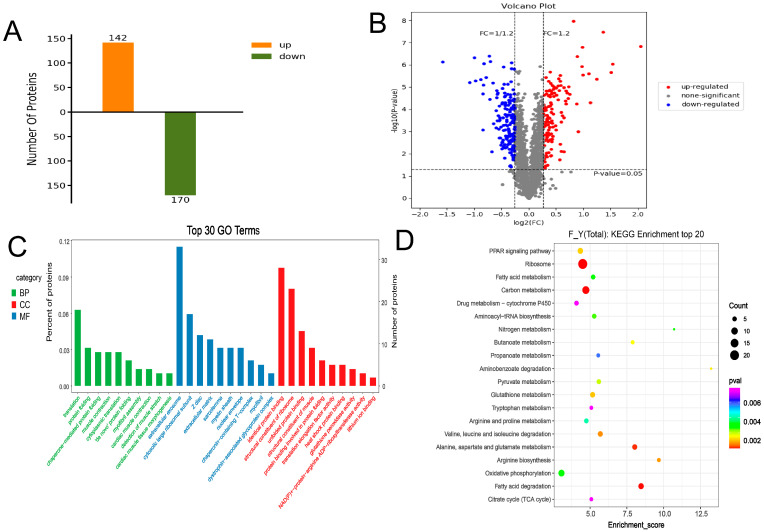
The number of differentially abundant proteins in (control/experiment) Group G vs. Group HF comparisons. (**A**) Total number of DAPs in the two groups, (**B**) Volcano plot of DAPs, (**C**) GO term enrichment for DAPs in the Group G vs. Group HF comparisons, (**D**) KEGG term enrichment for DAPs in the Group G vs. Group HF comparisons.

Liquid chromatography-tandem mass spectrometry (LC-MS/MS) analysis produced 278,259 total spectra; 62,127 spectra; 15,083 peptides; 10,083 unique polypeptides; and 1902 identified proteins (Figure 2A). Overall, >70% of the proteins contained at least two peptides (Figure 2B), and 97.7% of the proteins had a mass of ≥10 kDa (Figure 2C), thereby indicating data quality was good. In addition, 54% of the sequences had coverage distributions of >10% (Figure 2D), indicating the high quality of the proteomic data. We obtained 312 DAPS (142 upregulated and 172 downregulated proteins) in the two groups (Group G vs. Group HF; Figure 3A,B).

The GO enrichment analysis was performed to identify significantly enriched GO terms among the DAPs. At the proteomic level, DAPs detected in the Group G vs. Group HF comparison were significantly (*p* < 0.05) enriched for 169 GO terms (Table S3) and were mainly distributed in the terms Z disc, unfolded protein binding, myofibril, muscle contraction, and cytosolic large ribosomal subunit (Figure 3C). The KEGG enrichment analysis was conducted to identify significantly enriched KEGG terms among the DAPs. At the transcriptomic level, DAPs detected in the Group G vs. Group HF comparison were significantly (*p* < 0.05) enriched for 35 KEGG terms (Table S3) and were mainly distributed in the terms ribosome; FA degradation; alanine, aspartate, and glutamate metabolism; PPAR signaling pathway; and FA metabolism (Figure 3D). Three KEGG terms (PPAR signaling pathway) were enriched at two levels, thus revealing that some identified DEGs/DAPs might impact meat quality.

### 3.4. Correlation between Transcriptome and Proteome

**Figure 4:**
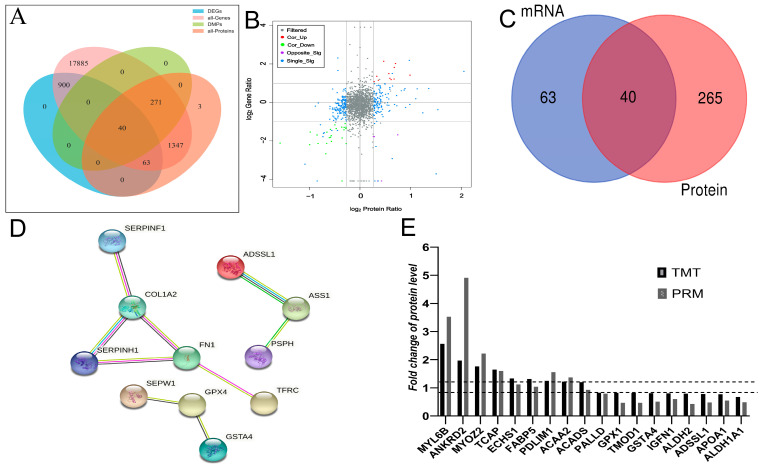
Correlation analysis of the DEGs and DAPs. (**A**) Venn diagram of all detected genes, DEGs, all detected proteins, and DAPs for Group G compared with Group HF (**B**) A correlation between transcripts (*y*-axis) and proteins (*x*-axis) in the Group G vs. Group HF comparison. Note: The abscissa is the log2 value of DAPS, and the ordinate is the log2 value of DEGs. Gray dots represent filtered non-differenced proteins (genes), and red dots (Cor-Up) represent differential proteins (genes) that are also upregulated. The green dots (Cor-Down) represent differentially expressed proteins (genes) simultaneously downregulated. The purple dots (Opposite_Sig) represent the DAPs (genes), which exhibited the opposite trend. The blue dots (Single_Sig) represent differential proteins (genes) that are differentially expressed at only one protein or mRNA level. (**C**) Venn diagram shows the overlap of DEGs and DMPs. (**D**) PPI analysis reveals the relationship between target DEGs and DAPs. (**E**) Validation of TMT by PRM analysis.

As shown in Figure 4A, of the 1003 DEGs, 963 DEGs had no corresponding protein in the proteomic data. This was possibly due to the low sensitivity of proteomic detection. In the proteomic data, the abundance of 63 DEGs of protein products exhibited no change between the two groups (i.e., they were not DAPs). In total, 40 DEGs/DAPs have changed in the transcription and protein levels (Figure 4A,C). The transcriptome and proteome data are from the same sample. Thus, DEGs and DAPs were found to exhibit the same expression patterns and facilitated each other in confirming gene expression regulation. The protein expression pattern in quadrants 3 and 7 was the same as that in the transcript (Figure 4B). Among the 40 DEGs/DAPs, 16 upregulated DEGs/DAPs and 24 downregulated DEGs/DAPs were located in the same expression trend quadrant (3 and 7), respectively. To ensure a more intuitive and clearer interaction of these DEGs/DAPs with the same expression patterns, the PPI network analysis was conducted using the STRING-db server. The more lines between two genes/proteins, the greater the chance of that interaction. The results revealed that DEGs/DAPs were closely related, and COL1A2 (degree = 10) corresponded to critical nodes in the network, followed by SERPINF1, FN1, and SERPINH1 (Figure 4D). In total, 18 DAPs were selected to validate the DAPs identified through TMT·LC-MS/MS analysis. PRM validated the -proteomic data at the protein level. Eighteen differentially rich proteins were randomly selected in the proteome. The PRM analysis demonstrated that the expression trends of all proteins, except ·ACADS, were similar to those observed through TMT, which supported the confidence of the proteomic data (Figure 4E and Table S4)

## 4. Discussion

Yaks are raised in highly harsh environments all year round, and every year, there is a dry season period. However, the recent expansion of the yak population has severely degraded the grassland, and the growth performance of yaks has reduced significantly [19]. In our experiment, compared with the grazing group, the average daily gain and average net body weight of yaks in the HF group increased by 0.34 and 70.34 kg, respectively. The results showed that house-feeding could significantly improve the production performance of yaks and generate more economic benefits.

The tenderness of meat is an essential indicator of the quality of meat products. It is a vital factor for evaluating meat quality by consumers and affects meat consumption. Tenderness is mainly determined by connective tissue content, followed by muscle fiber diameter, sarcoplasmic protein content, and intramuscular fat (IMF) content [20,21]. Yaks are grazed and maintained for a long period and slaughtered at an older age, which results in poor tenderness of yak meat and restricts its market acceptance. Many studies have shown that house feeding can improve the IMF content of LD muscle, reduce shear force, and improve the quality of yak meat [7,22]. Glycogen and lactic acid content in the muscle affect the muscle pH value. Therefore, pH is also among the crucial factors affecting meat quality. After slaughter, muscle glycogen produces lactic acid through anaerobic glycolysis, and the pH of the muscle decreases with continuous lactic acid accumulation. This decreased pH value may inactivate u-calpain, reducing myofibrillar protein decomposition and, in turn, affecting meat tenderness and color [23]. The pH of cattle meat decreased faster than that of buffalo meat within 48 h after slaughter, and buffalo meat was more tender than yellow beef meat, presumably because of the higher pH and higher levels of protease activity, which increased the proteolysis rate, ultimately lowering shear force and increasing meat tenderness [24]. As shown in Table 3, the pH value decreased more slowly in the HF group than in the G group, and the shear force was also significantly lower in the HF group than in the G group. The aforementioned results indicate that effective feeding can improve the tenderness of yak meat. The shear force of the LD muscle was significantly lower in the HF group than in the G group. Thus, the HF group could change the composition of LD muscle fiber types and increase the intermuscular fat content, thus improving meat tenderness in yaks.

RNA-Seq and TMT proteomics offer advantages such as high resolution, good quantitation, and deep coverage [25,26]. The combined analysis of proteomics and transcriptomics allows the determination of critical information about meat quality and the growth and development of animals. However, until now, little is known about meat quality and the growth and development of yaks at different feeding regimes. In the transcriptomic analysis, 15,242 genes were identified in the HF and G groups through the transcriptome. In total, 1003 DEGs (387 upregulated and 616 downregulated genes) were differentially expressed in the two groups. GO analysis of the DEGs indicated that these genes identified during the comparison were involved in several important biological functions and processes, such as response to denervation involved in the regulation of muscle adaptation (FBXO32, MYOG, TRIM63, and HDAC4); positive regulation of myotube differentiation (MAML1, MAMSTR, MYOG, and SMYD1); and lipopolysaccharide-binding (SPON2, TLR4, LOC102281707, LOC102268707, and SCARB1). KEGG pathway enrichment revealed that DEGs could be significantly enriched in the AMPK signaling pathway, FoxO signaling pathway, PPAR signaling pathway and glycolysis/gluconeogenesis pathway. Thus, the differences in the quality and growth of yaks at different feeding levels were caused not by individual factors but by the combined effects of multiple genes and pathways. FoxO plays a crucial role in animal growth and development, cell differentiation, metabolism, apoptosis, and immunity through transcriptional regulation and signal transduction [27]. Studies have shown that FoxO1 in muscle fiber-type transition and myoblast differentiation during muscle tube formation is closely related to the gene, and FoxO1 has already been shown to negatively regulate type I fibers [28]. Leptin regulates SIRT1 and FoxO3a expression and then affects the expression of key functional genes related to lipolysis, thus affecting meat quality [29]. Both STR1 and FoxO3 were significantly upregulated in the HF group than in the G group, suggesting that the improvement in meat tenderness observed with the house feeding mode was regulated by key genes in FoxO pathways. Glycolysis is a crucial energy pathway in cells during the postmortem period, and the glycolysis rate is influenced by muscle fiber type. The gene expression of myosin heavy chain 2 9 (MYH2) in the HF group was higher than that in the G group (log2FoldChange = −0.88, *p* = 0.096), while MYH1 (−0.01, *p* = 0.980), MYH7 (−0.03, *p* = 0.962), and MYH4 (−0.31, *p* = 0.517; Table S5). The difference in multiples of difference was considerably greater than that of MYH2, indicating that under the action of glycolysis, type IIB fibers in the HF group were transformed into IIA. The study found that type IIA was positively correlated with muscle tenderness, and an increase in the proportion of type IIB muscle fibers leads to the promotion of a rapid drop in muscle pH and a reduction in meat tenderness [30,31]. This is consistent with the results of our experiment. The shear force was smaller, and PH24h was higher in the HF group than in the G group (Table 3). In the glycolysis/gluconeogenesis pathway, phosphoglycerate mutase 2 (PGAM2) deserves our attention. PGAM encodes crucial enzymes in the glycolysis pathway, which can catalyze 3-phosphoglycerate into 2-phosphoglycerate [32]. Mammals have two types of dPGAM, including dPGAM-B (PGAM1) and dPGAM-M (PGAM2). PGAM2 is mainly expressed in the myocardium of skeletal muscle and participates in the glycolysis process in the skeletal muscle [33,34]. The study found that PGAM2 affects all stages of skeletal muscle growth and is also associated with daily gain and slaughter traits [35]. Therefore, PGAM2 not only plays a vital role in glycolysis but also is closely related to meat quality traits and growth traits. In this experiment, the final body weight, average daily gain, and carcass weight were significantly higher in the HF group than in the G group (Table 2). The differential expression of PGAM2 is speculated to affect the growth traits of yaks.

In both groups, 1902 proteins were detected. Compared with the G group, 312 DAPs were detected in the HF group. The muscle proteomic difference in the number of DAPs between the two groups was 16.4%. Correspondingly, some essential terms and pathways were significantly enriched among the DAPs, including positive regulation of myotube differentiation; collagen fibril organization; Wnt-activated receptor activity; alanine, aspartate, and glutamate metabolism; glutathione metabolism; and PPAR signaling pathway. Interestingly, DAPs/DEGs were significantly enriched in the PPAR pathway at both transcriptional and protein levels. The PPAR signaling pathway is crucial for FA metabolism, sterol metabolism, and adipogenic differentiation [36]. PPARs are a class of nuclear transcription factors activated by ligands, which can be activated by FAs and their metabolites and belongs to the receptor superfamily.

PPARa, PPARβ, and PPARγ subtypes are currently involved in the PPAR signaling pathway [37]. In addition, RXR is another core element of this pathway. When upstream FAs and other ligands activate PPARs, RXR binds to them, forms heterodimers, and regulates gene expression by binding to PPRE, a specific DNA response element located upstream of lipid metabolism- or adipogenic differentiation-related genes [38]. This leads to complete signal transduction of the whole signaling pathway and the regulation of downstream lipid metabolism, adipocyte differentiation, and other biological functions. In the two experimental groups, several key DAPs and DEGs in the FABP family were also significantly enriched in the PPAR pathway. FA-binding protein (FABP), a member of the intracellular lipid-binding protein superfamily, mainly exists in the cytoplasm of vertebrates and invertebrates. It is a small-molecule protein in cells that is abundant in most tissues. FABP can participate in the transport of intracellular FAs and is vital for the uptake, transport, and metabolism of intracellular long-chain FAs [39,40]. FABP3 can transport FAs from the cytoplasmic membrane to esterification and oxidation sites, allowing them to enter the mitochondrial energy metabolism system, where FAs are oxidized and decomposed and eventually generate adenosine triphosphate (ATP). This ATP provides energy for myocardial contraction. FABP3 and FABP1, two FA-binding genes, have been proposed as candidates for IMF accumulation in mammals [41]. *FABP3* polymorphic loci can affect the IMF deposition capacity [42,43], thus affecting meat quality. In this experiment, the energy level was considerably higher in the HF group than in the G group, so the energy consumed by the feeding group was converted into IMF, thereby affecting the taste of yak meat.

Determining the PPI network provides a conceptual framework for a clearer and better understanding of the functions of DEGs and DAPS. Through the PPI network, a close relationship was observed between 11 shared DEG/DAPS (Figure 4D). The central network comprises collagen biosynthesis-related genes/proteins, such as collagen type I alpha 2 chain (COL1A2), and COL1A2 encodes cartilage matrix protein type I collagen, consists of three identical polypeptide chains that form fibers and is an essential structural component of the extracellular matrix [44]. Type I collagen, a member of the collagen family, has the typical characteristics of collagen. It is the main protein component in bone and most connective tissues, and it regulates the formation, differentiation, and stiffness of muscle tissue fibers. The type I collagen molecule consists of two identical AL chains folded into a left-handed triple helix structure: the A2 chain encoded by *COL1A1* and the A2 chain by *COL1A2* [45]. Collagen is the most crucial protein with a role in connective tissue and an essential factor affecting muscle quality [46]. The tenderness of fish [47] and pig [48] muscle is negatively correlated with the collagen content in the muscle, and the higher the collagen content, the lower the tenderness. In our experiment, the COL1A2 protein expression level was significantly downregulated in the HF group, and the meat tenderness was higher in the feeding group than in the grazing group, indicating that collagen content was negatively correlated with meat quality, which was consistent with previous study findings.

## 5. Conclusions

In our study, the transcriptomic and proteomic profiles of grazing and in-house-fed yak groups (were compared, presenting the significance of FA metabolism, regulation of the steroid metabolic process, and FA degradation in meat quality. The 1003 DEGs and 312 DAPs were identified according to the transcriptomic and proteomic analysis results; these are enriched in the PPAR pathway. The energy level was considerably higher in the feeding group than in the grazing group. Thus, this energy consumed by the feeding group was converted into IMF, thereby affecting the taste of yak meat. The PPI network analysis revealed COL1A2 as a hub gene that might affect yak meat tenderness. These results expand our knowledge of yak meat quality and offer adequate information and more evidence for understanding the biological mechanisms underlying meat quality traits.

## Figures and Tables

**Table 1:** Basic Diet Composition of Group HF.

Item	Content (%)
Corn	48.53
Wheat Bran	6.22
Rapeseed Cake	7.25
Baking Soda	0.25
Dairy Salt	0.25
4% Premix Compound	1.5
Oat Hay	22
Alfalfa Hays	14
Total	100

**Table 2:** Growth performance of yaks in different feeding systems.

Carcass Characteristics	Group G	Group HF	*p*-Value
Initial body weight/kg	208.33 ± 12.53	215.67 ± 21.83	0.6549
Final body weight/kg	306.83 ± 24.34 B	377.17 ± 15.77 A	0.0010
Average daily gain/kg	0.52 ± 0.12 B	0.86 ± 0.15 A	0.0017
Carcass weight/kg	124.28 ± 8.77 B	208.04 ± 8.75 A	<0.001
Net meat weight/kg	94.73 ± 4.55 B	176.23 ± 10.61 A	<0.001
Bone weight/kg	31.08 ± 1.96 b	27.17 ± 2.46 a	0.012
Slaughter rate/%	42.49 ± 10.24 B	55.26 ± 14.25 A	<0.001
Meat-bone ratio	3.46 ± 0.09 B	5.68 ± 0.29 A	<0.001
Eye muscle area/cm^2^	57.89 ± 8.83 A	76.64 ± 10.45 B	<0.001

The average daily gain in grazing yak is negative. Different lowercase letters a and b in the same peer indicate *p* < 0.05, and capital letters A and B indicate *p* < 0.01 (the same below).

**Table 3:** Effects of fattening on meat quality in LD muscle of yaks.

Meat Quality	Group G	Group HF	*p*-Value
L* 45 min	6.32 ± 0.80	6.98 ± 1.27	0.305
a* 45 min	28.12 ± 2.32	29.29 ± 2.49	0.418
b* 45 min	5.67 ± 1.04	6.42 ± 1.38	0.311
L * 24 h	7.61 ± 0.99	8.86 ± 1.20	0.054
a* 24h	32.68 ± 0.91	33.23 ± 1.19	0.385
b* 24 h	9.94 ± 1.18	8.62 ± 0.81	0.047
pH 45 min	6.89 ± 0.32 A	6.34 ± 0.24 B	0.007
pH 24 h	5.49 ± 0.25 B	6.03 ± 0.28 A	0.005
Cooking loss (%)	17.82 ± 3.84 B	27.21 ± 1.70 A	<0.001
Drip loss (%)	15.06 ± 2.30	17.27 ± 3.12	0.192
Shear force (N)	17.70 ± 1.51 A	14.69 ± 2.35 B	0.0025

## Data Availability

Data is contained within the article or Supplementary Material.

## Supplementary Materials

The following are available online at https://www.mdpi.com/article/10.3390/foods12020257/s1, Table S1: Genome map Rate. Table S2: Significantly enriched KEGG pathways and GO terms for RNA-seq. Table S3: Significantly enriched KEGG pathways and GO terms for TMT. Table S4: The PRM analysis results of validated proteins. Table S5: FPKM of myosin in RNA-seq.

## Abbreviations

TMT	Tandem Mass Tag
LD	*Longissimus dorsi*
DEGs	Differentially expressed genes
DAPs	Differentially abundant proteins
PRM	Parallel reaction monitoring
FA	Fatty acid
HPLC	High-performance liquid chromatography
FC	Fold change
KEGG	Kyoto Encyclopedia of Genes and Genomes
GO	Gene Ontology
PPI	Protein-protein interaction
LC-MS/MS	Liquid chromatography-tandem mass spectrometry
IMF	Intramuscular fat
FABP	FA-binding protein
MYH	Myosin heavy chain
PGAM2	Phosphoglycerate mutase 2
COL1A2	Collagen type I alpha 2 chain
PPAR	Peroxisome proliferator-activated receptor
FoxO	Forkhead box, sub-group O
FDR	False discovery rate
